# SSH adequacy to preimplantation mammalian development: Scarce specific transcripts cloning despite irregular normalisation

**DOI:** 10.1186/1471-2164-6-155

**Published:** 2005-11-08

**Authors:** LC Bui, RD Léandri, JP Renard, V Duranthon

**Affiliations:** 1 UMR Biologie du Développement et de la Reproduction. INRA 78350 Jouy en Josas. France

## Abstract

**Background:**

SSH has emerged as a widely used technology to identify genes that are differentially regulated between two biological situations. Because it includes a normalisation step, it is used for preference to clone low abundance differentially expressed transcripts. It does not require previous sequence knowledge and may start from PCR amplified cDNAs. It is thus particularly well suited to biological situations where specific genes are expressed and tiny amounts of RNA are available. This is the case during early mammalian embryo development. In this field, few differentially expressed genes have been characterized from SSH libraries, but an overall assessment of the quality of SSH libraries is still required. Because we are interested in the more systematic establishment of SSH libraries from early embryos, we have developed a simple and reliable strategy based on reporter transcript follow-up to check SSH library quality and repeatability when starting with small amounts of RNA.

**Results:**

Four independent subtracted libraries were constructed. They aimed to analyze key events in the preimplantation development of rabbit and bovine embryos. The performance of the SSH procedure was assessed through the large-scale screening of thousands of clones from each library for exogenous reporter transcripts mimicking either tester specific or tester/driver common transcripts. Our results show that abundant transcripts escape normalisation which is only efficient for rare and moderately abundant transcripts. Sequencing 1600 clones from one of the libraries confirmed and extended our results to endogenous transcripts and demonstrated that some very abundant transcripts common to tester and driver escaped subtraction. Nonetheless, the four libraries were greatly enriched in clones encoding for very rare (0.0005% of mRNAs) tester-specific transcripts.

**Conclusion:**

The close agreement between our hybridization and sequencing results shows that the addition and follow-up of exogenous reporter transcripts provides an easy and reliable means to check SSH performance. Despite some cases of irregular normalisation and subtraction failure, we have shown that SSH repeatedly enriches the libraries in very rare, tester-specific transcripts, and can thus be considered as a powerful tool to investigate situations where small amounts of biological material are available, such as during early mammalian development.

## Background

Molecular analysis during the early period of embryonic development has long been prevented in mammals because of the scarcity of biological material, whatever the species considered. In recent years, however, technical improvements in the analysis of messenger RNAs from tiny amounts of cells have revealed the complexity of the genome expressed during the preimplantation period [1,2]. This complexity has been highlighted in recent publications which reported the isolation of new sequences in different mammalian species [3-6]. Although the transient and tissue-specific expression of such sequences during later development cannot formally be ruled out, they are more likely to be specifically expressed during the preimplantation stages, which has thus prevented their identification until now [7]. This underlines the need for dedicated transcriptomic tools to investigate these developmental stages. Such tools now exist for studies in mice [8], but still have to be developed for other species. Of the different strategies available to establish cDNA libraries, Suppression Subtractive Hybridisation (SSH) [9] is an efficient and widely-used PCR-based method to obtain subtracted libraries and isolate differentially expressed genes. The procedure involves two successive tester-driver hybridisation steps, the first of which induces a normalisation of tester-specific molecules, thus allowing the subsequent cloning of rare, tester-specific transcripts. Because SSH can be initiated using PCR-amplified cDNAs, it seems particularly well-suited to mammal preimplantation stage embryos which contain only a few tens of picograms of mRNAs. Furthermore, because SSH does not require previous knowledge of gene sequences, it may also be suitable for species where only a small number of sequences are available in databases.

Although it has already been used to get insight into early embryo transcriptome [10,4,6,11], SSH performance in this area has not been widely documented. This is because authors usually establish only one dedicated library and provide information restricted to a few biologically relevant clones isolated from this library. Because we were interested in the more systematic establishment of SSH libraries dedicated to the analysis of early mammalian embryo development, we designed a procedure involving exogenous reporter transcripts that mimic either tester-specific or tester-driver common transcripts to enable the large scale assessment of the quality of such libraries. This procedure was applied to four independent libraries and provided information on their quality and the repeatability of the SSH procedure applied to early-stage embryos. These data were further validated by the sequencing and clustering of about 1600 clones isolated from one of these libraries. Our results show that when applied to preimplantation mammal embryos, the cDNAs of which had been pre-amplified using the SMART (Clontech patent) procedure, SSH-generated libraries repeatedly provided access to very scarce, tester-specific transcripts, despite irregular normalisation and some subtraction failures.

## Results and discussion

Three of the libraries we established aimed to analyse embryonic genome transcriptional activation in rabbit and cattle (the so-called rab1, rab3 and bov1 libraries), while the fourth library (rab2) was designed for studies on early cell differentiation at the blastocyst stage in the rabbit.

**Figure 1:**
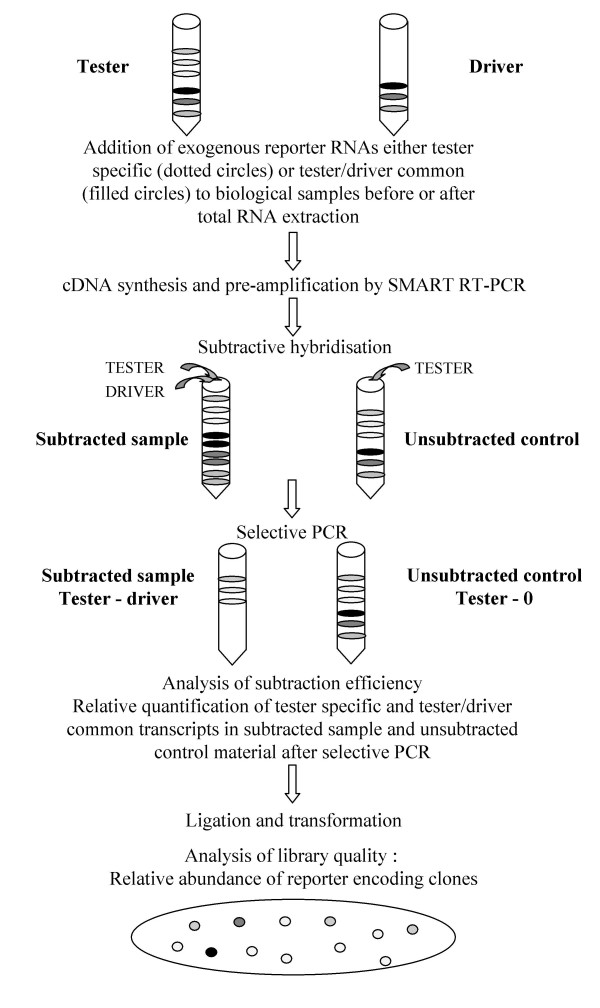
Experimental procedure designed to check SSH efficiency.

**Figure 2:**
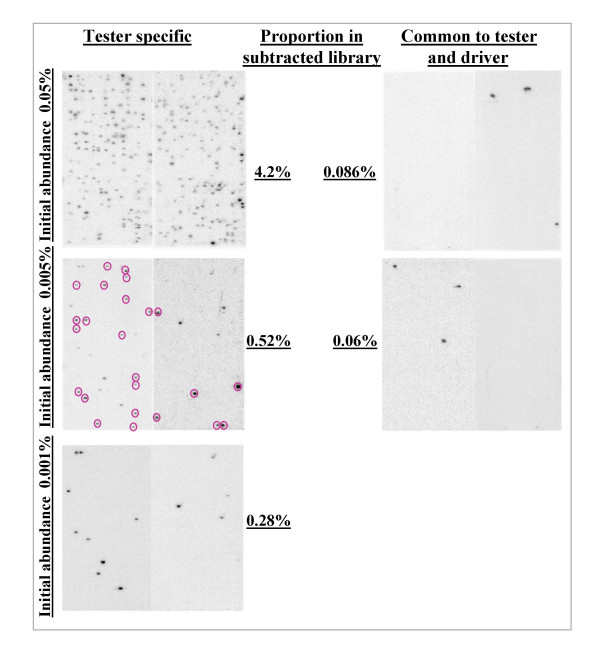
**Abundance of reporter-transcript encoding clones in the bov1 subtracted library**. Screening of bov1 bacteria macroarray with reporter RNA probes. Because of independent repeated hybridisations (of some membranes), only encircled clones on the left hand side (middle row) membrane should be considered as hybridising with the relevant probe.

In order to achieve a broad appraisal of the quality of the libraries, we decided to array several thousand clones from each library and analyse the abundance of tester-specific and tester/driver common transcripts in the subtracted libraries after bacteria transformation. However, neither strict tester-specific transcripts nor tester/driver common and equally expressed transcripts are identified at these stages in bovine and rabbit embryos, so we introduced exogenous *A. thaliana *transcripts into our biological material prior to pre-amplification and subtraction (see Fig. 1). We screened 768, 4608, 2683 and 4608 clones from the rab1, bov1, rab2 and rab3 libraries, respectively, for the presence of rare, tester-specific transcripts, using hybridisation with probes corresponding to the exogenous transcripts added. Whatever the lowest abundance of the reporter RNA (0.001% or 0.0005% of messenger RNAs), we found clones which encoded for the scarce, tester-specific transcript in the libraries. These clones represented more than 0.1% of the colonies (Table 1). This result was in agreement with the initial findings of Diatchenko's group [9], but disagreed with the results published more recently by Ji *et al*. [12] that suggest that only abundant targets (0.1% of messenger RNA) underwent efficient enrichment by SSH PCR, thus precluding the detection of transcription factors and receptors. Such divergent conclusions may have been due to differences in analytical sensitivity, because Ji *et al*. only considered subtracted cDNA smears after agarose gel electrophoresis [12], whereas we applied the hybridisation of specific, radiolabelled probes to thousands of bacterial colonies. We also found that exogenous reporter RNAs were differentially represented among the bacterial colonies, in line with their initial abundance in the tester material (Table 1 and Fig. 2). While clones encoding rare and moderately abundant tester-specific transcripts represented 0.2 to 0.5% of the clones in the libraries, abundant tester-specific transcripts were very frequently represented in the subtracted libraries (5 to 10 % of clones). These results thus show that rare and moderately abundant transcripts are roughly normalised by the SSH procedure while abundant transcripts are not. This is inconsistent with the conclusions reached by both Diatchenko *et al *[9] and Ji *et al *[12], suggesting that all or nothing differentially expressed cDNAs were enriched to a similar final level, irrespective of their initial concentration. Here again, these divergent conclusions could be explained by differences in the experimental procedures: during our study, reporter transcripts were added to a complex biological material containing both commonly and differentially expressed transcripts, whereas tester-specific reporter DNA were added to a common cDNA used as tester and driver material by Diatchenko [9] and Ji [12]. The hybridisation kinetics during our study were probably more representative of most biological situations. Moreover, two other experimental approaches allowed us to conclude that the unequalized abundance of exogenous transcripts in our libraries was representative of the behaviour of endogenous transcripts.

**Table 1:** Proportion of reporter transcripts encoding clones in the subtracted libraries

	**Library**	**Initial abundance (%)** Tester specific (**bold**) Tester/Driver Common (standard)	**Hybridised/analysed clones**	**Proportion in subtracted library (%)**
**Rare transcripts**	rab1	#	#	#
	bov1	**0,001**	**13/4608**	**0,28**
	rab2	**0,0005**	**3/2683**	**0,11**
	rab3	**0,0005**	**14/4608**	**0,3**

**Moderate transcripts**	rab1	**0,005**	**5/768**	**0,65**
		0,005	0/768	-
	bov1	**0,005**	**24/4608**	**0,52**
		0,005	3/4608	0,06
	rab2	**0,002**	**22/2683**	**0,82**
		0,002	0/2683	-
	rab3	**0,002**	**11/4608**	**0,24**
		0,002	2/4608	0,043

**Abundant transcripts**	rab1	**0,05**	**82/768**	**10,7**
		0,05	10/768	1,3
	bov1	**0,05**	**195/4608**	**4,2**
		0,05	4/4608	0,086
	rab2	**0,01**	**147/2683**	**5,48**
		0,01	0/2683	-
	rab3	**0,01**	**256/4608**	**5,55**
		0,01	25/4608	0,54

#: not introduced in library

-: no clone detected among the screened clones

First of all, we picked seven clones at random from the rab2 library that did not encode for exogenous transcripts, and analysed their abundance in the library by hybridising each radiolabelled insert to 2683 clones from the library. Three of them were present only once, the others (3L22, 3P11, 3I20 and 3C24) were found 29, 63, 152 and 362 times, respectively. The most abundant of these clones (3C24) thus represented 13.5% of the clones in the rab2 library. Its sequence corresponded to a fragment of rabbit mitochondrial 16S rRNA (nucleotide 1402 to 1899, Accession number AJ001588).

Secondly, we systematically sequenced 1920 clones from the rab2 library that did not hybridise to the 3C24 insert. From these clones, 1582 "good quality" sequences were assembled into 651 distinct contigs, of which 447 were singlet contigs. The depth of the 204 remaining contigs ranged from 2 (98 contigs) to 135 (1 contig). Only 14 contigs contained more than 10 sequences, two of them corresponding to *A. Thaliana *tester-specific exogenous transcripts (initial abundance 0.01 and 0.002%). These 14 contigs totalized 638 sequences. Surprisingly, the deepest contig – that containing 135 (8.5%) of the sequenced clones – encoded for the complete mitochondrial 16S rRNA, with a high prevalence of sequences bordering the 3C24 fragment. Finally, about 22% of the rab2 library clones encoded for this mitochondrial cDNA.

Sequence data validated the results obtained by hybridisation for tester-specific exogenous transcripts. We found 86/1582 (5.43%) clones encoding for the abundant reporter tester-specific transcript, 14/1582 (0.88%) clones encoding for the moderately abundant one and 6/1582 (0.38%) for the rare one, these results being in agreement with hybridisation results (Table 1). Sequencing also confirmed the results obtained for common transcripts in the rab2 library, since no sequenced clone corresponded to these exogenous transcripts (Table 1). However, "commonly expressed transcript" elimination seemed somewhat variable in the four libraries we analysed (Table 1). In the worst case (rab3 library, moderate transcript initial abundance), with respect to transcripts of the same initial abundance, we observed five-fold less representation in the library for the tester/driver commonly expressed transcript when compared with the tester-specific transcript (Table 1).

The normalisation failures observed for abundant exogenous reporter-transcripts probably reflected the results concerning endogenous transcripts: the coexistence of a majority of singlet contigs (68%) with a few, very deep contigs.

In order to obtain more information about highly redundant endogenous sequences in the libraries, we returned to the 3L22, 3P11, 3I20 and 3C24 transcripts. Their high level of representation in the library suggested that they encode for abundant transcripts, but the mitochondrial nature of 3C24 rendered tester-specific expression highly unlikely. We thus analysed the relative abundance of these four cDNAs in tester and driver unsubtracted materials. Semi-quantitative analysis (see methods) revealed that these four clones encode for very abundant cDNA in the tester material : they represented about 0.11, 0.6, 2.45 and 3.7% (for 3P11, 3I20, 3L22 and 3C24 respectively) of blastocyst cDNAs. They thus constituted a fourth category of transcripts that we did not mimic with the exogenous *A. thaliana *transcripts. Two of them (3P11 and 3I20) were tester-specific, but the others (3L22 and 3C24) were expressed in both tester and driver, with double their relative abundance in tester than in driver (3L22 and 3C24 representing respectively about 1.2 and 1.5% of morula driver cDNAs). It thus appears that very abundant tester-specific transcripts escape normalisation, whereas very abundant transcripts expressed in both tester and driver escape both subtraction and normalisation.

Under these conditions, the few deep contigs we obtained by sequencing the library could correspond to either abundant or very abundant tester-specific transcripts escaping normalisation, or to very abundant commonly expressed transcripts escaping both subtraction and normalisation. These normalisation (and possibly subtraction) failures resulted in a significant proportion of highly redundant sequences (638/1582, or 40%) in the library.

In view of the considerable concordance between sequencing and hybridisation data, we assumed that the addition of exogenous reporter transcripts and analysis of their representation in the library would ensure reliable monitoring of SSH performance. However, the very high representation of mitochondrial 16S rRNA in the library showed that the frequency we chose to mimic abundant transcripts underestimated the representation of certain transcripts in our biological material. 16 SrRNA was found to represent a high proportion (1.5 to 3.7%) of unsubtracted cDNAs, which could be correlated to the very rapid growth [13] and marked metabolic activity of the blastocyst in the rabbit species, which certainly requires strong mitochondrial activity during the early stages of development. Mitochondrial transcripts have been shown to represent as much as 23% of polyadenylated RNAs in mouse blastocysts [14]. Such quantitative data are not available in the rabbit.

Our results show that application of the SSH procedure to early development permits the isolation of scarce tester-specific transcripts despite irregular normalisation and some subtraction failures concerning very abundant transcripts.

Taken together, these data confirmed and extended previous reports showing that whatever the origin of the clones picked from SSH constructed libraries – random choice or selection by differential screening – some of them are heavily represented among the sequenced clones [[9,10,4,15], and [16]]. Since this unequalized representation has been reported in both SMART-preamplified-cDNA subtracted libraries [10,4,16] and non preamplified cDNA libraries [15], it should not be considered as a consequence of the cDNA preamplification step but rather as an intrinsic defect in the SSH procedure.

## Conclusion

This study showed that SSH libraries dedicated to early mammalian development are greatly enriched in tester-specific transcripts. They are only partially normalized, with transcript equalization being restricted to rare and moderately abundant transcripts. Very abundant transcripts common to both tester and driver may escape both normalisation and subtraction, giving rise to abundant background clones in these libraries. The differential expression of genes represented by redundant clones in subtracted libraries should therefore be checked very carefully.

These conditions were however compatible with the isolation of very rare tester-specific transcripts (0.0005% of messenger RNA) in the libraries. Under these conditions, SSH produced reproducible results in terms of rare stage-specific transcript isolation and can thus be considered as a tool of considerable potential when studying the onset of mammalian development.

## Methods

### Developmental stages used for the construction of the libraries

The following stages were used in tester-driver subtractions:

- for rabbit embryonic activation: early morulae (16–32 cell stage) – one cell stage (rab1 library) and early morulae – 4 cell stage (rab3 library).

- for cattle embryonic activation: early morulae – four-cell stage (bov1 library)

- for early cell differentiation in rabbit: blastocyst – late morulae (32–64 cell) (rab2 library).

### Embryo recovery

Cattle embryos were obtained by *in vitro *oocyte maturation, fertilization and embryo culture as described by Pavlok *et al*. [17] and Parrish *et al*. [18]. Four-cell and morulae stage embryos were recovered at 41 and 120 hours post-insemination respectively, from early two-cell-cleaved embryos picked up at 32 hours post-insemination.

For rabbit embryos, all tester materials contained embryos produced both *in vivo *and *in vitro*, whereas driver materials contained only embryos produced *in vivo. In vivo *one-cell, four-cell, early morulae, late morulae and blastocyst stage embryos were recovered at 19, 32, 50, 65 and 90 h post-coitum (h*pc*), respectively. *In vitro *embryos were recovered at the one cell stage (19 h post-coitum) from superovulated females treated as described by Henrion *et al*. [19]. They were cultured from the one cell stage onwards (19 h*pc*) until the early morula (58 h*pc*) and blastocyst (100 h*pc*) stages respectively in four different culture media: B2 medium (Laboratoire C.C.D.), B2 medium plus 2.5% foetal calf serum, ISM1–ISM2 sequential media (Medicult.) and G1–G2 sequential medium (JCD sa). In the latter two cases, the sequence used for embryo culture mimicked that used in human IVF in terms of the timing of genome transcriptional activation: embryos were cultured in the first medium until the 8 cell stage in ISM1 and until the early morula stage in G1, i.e. just before and just after the onset of embryonic genome transcription respectively, then transferred to the second medium until the stage of interest.

### RNA extraction

Total RNA was extracted from batches of embryos (n = 200 to 450 embryos) using the RNeasy Mini Kit (Qiagen, CA, USA) with a DNAse I treatment (37°C, 30 min). *Arabidopsis Thaliana *RNAs (Stratagene, Spike RNA 201, 204, 205, 208, and 209) were added as reporter exogenous RNAs at different concentrations, either specifically in tester material or in both tester and driver materials. These exogenous RNAs were added either before (rab1 and bov1 libraries) or after (rab2 and rab3 libraries) total RNA extraction.

### cDNAs amplification, SSH and PCR amplification of subtracted products

The tester to driver hybridisation steps in the SSH procedure require one hundred nanograms of tester and driver cDNA, whereas a preimplantation embryo only contains a few picograms of mRNAs. For this reason, we adopted the SMART PCR cDNA amplification procedure (SMART-PCR cDNA Synthesis Kit: Clontech, Palo Alto, CA, USA) starting from embryonic total RNA. The optimum numbers of PCR cycles, checked as suggested in the SMART-PCR kit protocol, were 22 (rab1), 20 (bov1 and rab2), 23 (rab3), respectively.

SSH was performed with the PCR Select cDNA Subtraction Kit (Clontech, Palo Alto, CA, USA). The first hybridisation was performed with 15 ng amplified tester cDNA and 450 ng amplified driver cDNA for 10 hours at 68°C. Following the first hybridisation, 100 ng of fresh denatured driver cDNA was added to the sample and a second hybridisation was performed at 68°C overnight. The final hybridisation sample was diluted in 200 μl 20 mM Hepes pH 8.3, 50 mM NaCl, 0.2 mM EDTA buffer. Selective PCR was performed according to the recommendations of the kit manufacturer, except that the cycle numbers in the first and nested PCR were modified as mentioned in the text.

### Checking "selective PCR"

In order to check subtraction efficiency after selective PCR, relative amounts of tester-specific and tester/driver common exogenous *A. Thaliana *transcripts were estimated in the subtracted and control unsubtracted cDNA populations. As suggested by the kit manufacturer, this was achieved by semi-quantitative PCR amplification (gradually increasing the numbers of PCR cycles, and an analysis of amplicon intensity after agarose gel electrophoresis and ethidium bromide staining). We therefore used primers specific to *A. Thaliana *transcripts:

5'-201 TGGGTTAAGGCTCAGGAATG

3'-201 GCCAAGTGAGTTGCCAAGTT

5'-204 AACACAATGGCTTTCGCTTT

3'-204 CAAAGCCATCAAGACAAACAAA

5'-205 TTATTAGCCGTGTGCCTGGT

3'-205 CTAGCAAACCAATGCCCTCA

5'-209 TTCTGTCAATGGAGGCAACA

3'-209 TGTCAAACCAGAGCTCACGA

### Establishment and analysis of subtracted libraries

PCR-amplified subtracted products (about 17 ng) were cloned into the pGEM-T-Easy vector (Promega) and 1/10 of the ligation volume was used to transform competent DH5α Max-Efficiency *E.coli *bacteria (Invitrogen). After overnight culture at 37°C, the colonies were picked and arrayed in 384 well plates. Replicates of these arrayed libraries were spotted onto nylon membranes (Hybond N+ Amersham) placed on agar plates, and grown up for 12 hours at 37°C. After bacteria denaturation and DNA fixation treatments, these "macroarrays" were hybridised with ^32^P radiolabelled probes corresponding to either exogenous RNAs or endogenous transcripts. Hybridisation was carried out overnight at 65°C in Church buffer [20].

**Sequence analysis and clustering **were performed as described in [21].

### Semi-quantitative analysis of endogenous transcript abundance in tester and driver materials

Varying amounts (100, 300 500 ng) of unsubtracted SMART-amplified tester (blastocyst) and driver (morula) cDNAs used to establish the SSH library were slot blotted on Brightstar TM-Plus membranes (Ambion). On the same membranes, various quantities (ranging from 0.01 ng to 6.25 ng) of cDNA inserts (3P11, 3C24, 3L22 and 3I20, respectively) were slotted. DNA inserts (3P11, 3C24, 3L22 and 3I20) were labelled by random priming (Rediprime TM II Amersham). Hybridization was performed in UltrahybTM buffer (Ambion), at 42°C, for 22 hours. Washings were performed twice in 2 × SSC, 0.1% SDS at 42°C for 10 minutes, then twice in 0.1 × SSC, 0.1% SDS, 42°C, for 30 minutes. Membranes were exposed to a phosphoscreen (Phosphorimager Amersham) for 6 hours, and hybridization signals were quantified using Imagequant (Amersham).

## Authors' contributions

Véronique Duranthon is the supervisor of Linh Chi Bui and Roger Dominique Léandri, two doctoral students in the laboratory who participated directly in the experimental work. Jean Paul Renard is Director of the Laboratory.
